# Micro-CT for Biological and Biomedical Studies: A Comparison of Imaging Techniques

**DOI:** 10.3390/jimaging7090172

**Published:** 2021-09-01

**Authors:** Kleoniki Keklikoglou, Christos Arvanitidis, Georgios Chatzigeorgiou, Eva Chatzinikolaou, Efstratios Karagiannidis, Triantafyllia Koletsa, Antonios Magoulas, Konstantinos Makris, George Mavrothalassitis, Eleni-Dimitra Papanagnou, Andreas S. Papazoglou, Christina Pavloudi, Ioannis P. Trougakos, Katerina Vasileiadou, Angeliki Vogiatzi

**Affiliations:** 1 Hellenic Centre for Marine Research (HCMR), Institute of Marine Biology, Biotechnology and Aquaculture (IMBBC), P.O. Box 2214, 71003 Heraklion, Crete, Greece; ceo@lifewatch.eu (C.A.); chatzigeorgiou@hcmr.gr (G.C.); evachatz@hcmr.gr (E.C.); magoulas@hcmr.gr (A.M.); cpavloud@hcmr.gr (C.P.); kvasileiadou@hcmr.gr (K.V.); 2 Biology Department, University of Crete, 70013 Heraklion, Crete, Greece; 3 LifeWatch ERIC, 41071 Seville, Spain; 4 First Department of Cardiology, AHEPA University Hospital, Aristotle University of Thessaloniki, 54636 Thessaloniki, Greece; stratoskarag@gmail.com (E.K.); anpapazoglou@yahoo.com (A.S.P.); 5 Department of Pathology, Faculty of Medicine, Aristotle University of Thessaloniki, 54124 Thessaloniki, Greece; tkoletsa@auth.gr; 6 Medical School, University of Crete, 71003 Heraklion, Crete, Greece; makriskon01@gmail.com (K.M.); mavro@imbb.forth.gr (G.M.); molgrad320@edu.biology.uoc.gr (A.V.); 7 IMBB, FORTH, 70013 Heraklion, Crete, Greece; 8 Department of Cell Biology and Biophysics, Faculty of Biology, National and Kapodistrian University of Athens (NKUA), 15784 Athens, Greece; epapanagnou@biol.uoa.gr (E.-D.P.); itrougakos@biol.uoa.gr (I.P.T.)

**Keywords:** micro-computed tomography, scanning electron microscopy, optical microscopy, confocal laser scanning microscopy

## Abstract

Several imaging techniques are used in biological and biomedical studies. Micro-computed tomography (micro-CT) is a non-destructive imaging technique that allows the rapid digitisation of internal and external structures of a sample in three dimensions and with great resolution. In this review, the strengths and weaknesses of some common imaging techniques applied in biological and biomedical fields, such as optical microscopy, confocal laser scanning microscopy, and scanning electron microscopy, are presented and compared with the micro-CT technique through five use cases. Finally, the ability of micro-CT to create non-destructively 3D anatomical and morphological data in sub-micron resolution and the necessity to develop complementary methods with other imaging techniques, in order to overcome limitations caused by each technique, is emphasised.

## 1. Introduction

In the past few decades, a great number of imaging techniques such as micro-computed tomography (micro-CT), scanning electron microscopy (SEM), conventional light microscopy (LM), and confocal laser scanning microscopy (CLSM) have been developed and used in biological and biomedical studies. The continuous technical improvements in terms of detectors, system design, speed, sensitivity, resolution, computational analysis, and side effects of applied energy have been crucial for the advancement of biology and biomedicine [1].

The demand for non-destructive, fast, and reliable imaging methods in life sciences [2] as well as the need for high-resolution three-dimensional (3D) imaging for revealing the tissue organisation within a whole organism topography [3] have led to the use of micro-CT in several cases. Over the years, micro-CT has invaded life sciences and has been employed widely in many different aspects from medicine to ecology. Micro-CT as a non-destructive and high-resolution imaging technique can be used complimentarily with other imaging techniques [4] in an attempt to integrate the visual results of the different methods and, thus, reach more solid scientific conclusions. The intrinsic physical limitations of each imaging technique have led to more comprehensive approaches by the combination of several techniques, compiling a broader range of information from cell and tissue-level histomorphology to three-dimensional structures [5,6].

The aim of this study is to compare the micro-CT technique with other commonly applied imaging techniques in biological and biomedical fields. This review attempts to present a general overview of the pros and cons of several imaging techniques (micro-CT, optical microscopy, scanning electron microscopy, and confocal microscopy), across different biological and biomedical use cases to provide guidance to users for the selection of the optimum combination of techniques for particular scientific questions.

## 2. Imaging Techniques

### 2.1. Micro-Computed Tomography

Micro-computed tomography (micro-CT) is a non-destructive imaging technique based on X-rays that allows the rapid digitisation of samples in three dimensions, and it has the ability to visualise the interior and exterior characteristics of a sample. The introduction of computed tomography (CT) in the 1970s [7] was an imaging revolution as for the first time the 3D structure of a sample could be revealed [8]. The continuous technical developments in CT scanners led to the invention of X-ray micro-CT scanners with resolution at sub-micron levels.

The main principle of micro-CT is the generation of a series of radiographs, which are called projection images, of a rotated sample that is placed between an X-ray source and an X-ray detector. Subsequently, the acquired projection images are reconstructed into cross-section images using reconstruction algorithms (for details in image acquisition workflow, see [9]).

In biomedical research, micro-CT has been used in a wide range of scientific fields such as musculoskeletal, neurological, cardiorespiratory, gastrointestinal research, and longitudinal studies for treatment effects (e.g., [10,11,12,13,14,15,16,17]). In recent years, it has been used also in biological fields such as taxonomy, ecology, and developmental research (e.g., [18,19,20]).

The non-destructive nature of micro-CT has facilitated its combination with other techniques such as histology [20]. According to Herdina et al. [6], correlating micro-CT 3D images and 2D sections with specialised histomorphological techniques enables the identification of histological structures and the creation of a dataset that can be used for further studies. Moreover, it can be used in rare and valuable samples, such as natural history museum specimens, by creating “cybertypes”, which are virtual representations of the type materials [6,9,18,20]. Especially, the in vivo micro-CT scanners give the ability to scan the same specimen in different time points (e.g., [21]), which is very important for longitudinal studies. Micro-CT also gives the ability for 3D analysis such as density estimation, porosity, structure thickness, and morphometric analysis [9].

Although dense structures such as bones can be visualised without any specific preparation [22], a limitation of micro-CT scanners is the low contrast of soft tissues due to low X-ray absorption, which can be overcome using contrast agents [23,24] (for a comprehensive list of contrast agents, see [9]). The choice of the most appropriate contrast agent (or whether a contrast agent should be used at all) depends also on the sample and possible restrictions of its use. For example, valuable material of taxonomic specimens should probably not be stained. This is due to the fact that stains may alter the characteristics of the tissues, and the removal of staining agents has not yet been explored enough to be safely used on unique and valuable specimens [25,26,27].

Micro-CT can be considered as a fast technology compared to other techniques that may require weeks to be completed, such as histology, as the scanning duration may vary from minutes to hours depending on the scanning parameters [28]. However, a great magnification and resolution can increase the scanning duration. A high scanning duration leads to higher radiation doses, which need to be considered in longitudinal studies such as oncological studies using in vivo micro-CT scanners [24,29]. Furthermore, the potential damage of the genetic material due to X-ray radiation should be taken into account, especially in natural history museum specimens, although recent studies indicated that X-rays did not provoke any damage to the specimens DNA [18,30,31]. However, the use of synchrotron tomography can overcome these limitations, as this technology can create volumetric datasets in sub-micron resolution in a few minutes and with a greater field of view [32].

Another limitation of this technique is that the original colours of the sample cannot be represented. Original colours are important in several cases such as taxonomy, where they may be used as characters for species identification [33], and ecology, where they may be used as environmental stress indicators [34,35]. However, the development of new techniques combining micro-CT with digital cameras enables the digitisation of both the internal structures and the natural surface colours [36]. The size of the structures is also another factor that needs to be considered, as structures below a certain size cannot be detected by micro-CT [9], and a technique with a greater resolution (e.g., scanning electron microscopy) may be more appropriate. The long-term storage of microtomographic data can also be an issue because of the large size of the data on disks. For example, the micro-CT datasets produced for the case studies presented in Section 3 vary from 30 to 300 GB.

In conclusion, micro-CT is a powerful imaging technique for multiple biological and biomedical applications. It has the ability to visualise the internal features of a sample in a non-destructive manner, and it is a fast technology that can achieve great resolution. A combination of different imaging techniques through the fusion of the different datasets could overcome any possible drawbacks [29].

### 2.2. Optical Microscopy

Optical microscopy (OM) is one of the oldest technologies used in biology and life sciences. It is widely used in a great variety of applications; therefore, it is a continuously developing field, proving new potentials for science and medicine. The first microscopes appeared in their present form in the 17th century, and they are the simplest devices of microscopy: the light passes through a series of lenses and gives a magnified image of the object. The examination of the samples is performed directly by the user through eyepieces [37,38]. Optical microscopes are categorised based on their operational mode to the brightfield microscopes where the light is transmitted through the object and the polarised light microscopes where the light is reflected from the surface of the object [37,39,40]. Technology allowed the advancement of microscopy by using cameras to capture digital images of the samples. Using digital imaging in microscopy has facilitated the measuring of samples or photo treatment to enhance images [38].

Similarly to optical microscopy, stereoscopy is also using light to offer a magnified representation of the specimen but with a different depth of field. The difference from the microscope as the perspective is given by merging the two reflections of the sample as seen by each eye [37]. Thus, the user has a better perspective of the geometry of the observed object.

Light microscopy is widely used in biological and medical sciences because it offers the potential to examine the structure and the function of a sample by direct observation and in many cases by non-invasive method [39,41]. However, despite the great advances, the method has serious limitations. Even to the microscopes/stereoscopes with optical systems of high quality and resolution (up to few μm), the features of the specimen can always affect the image attributes. The shape and the texture of the object can influence the resolution and create an unclear representation causing problems in the analysis [41]. Another difficulty is that with optical microscopy, the examination of the sample is limited on the surface [42] (however, for exceptions, see Section 2.3). Therefore, the method cannot be used in cases where the analysis requires an internal view of the sample, while at the same time, the sample should remain complete, or it is impossible to be dissected.

In many cases in biological and medical studies, the combination of optical microscopy with micro-CT is applied. This practice is common in taxonomic studies e.g., the work of [43], where the authors are describing *Metanephrocerus groehni* and *M. hoffeinsorum* flies that were enclosed in amber. Representations produced by the micro-CT revealed phenotypic characteristics that changed the taxonomy within the family of Pipunculidae. Another application is in clinical studies where the methods were combined to quantify the radial geometry of the renal cortical vasculature in rats and rabbits [44]. The authors claim that a combination of micro-CT and optical microscopy is necessary to determine the spatial relationships of vessels with different diameters. In medical studies, the combining approach has been proven very efficient to detect cochlear trauma caused by cochlear implantation [45]. The study concludes that a combination of 2D imaging, 3D imaging, and histology is necessary in order to have reliable results regarding the trauma.

Light microscopic histology is commonly used to visualise the internal structure by the sectioning of tissues [3]. Imaging and histology are two morphological techniques that are interrelated [46]. Likewise, histology and histopathology are interlinked disciplines [47]. Histology is the examination of normal cells and tissue (at the cellular level), while histopathology is the study of diseased tissues [48]. As such, histopathology focuses on whole organs or small samples of larger tissues [48].

The basis of histology relies on the construction of the first microtome, which could be used to section animal tissues, and on the usage of paraffin wax for infiltration and support during sectioning [47]. Additionally, the dissected tissues can be submerged in certain chemical substances, i.e., fixatives, to preserve the morphologic and chemical characteristics of the specimen, while preventing digestion by enzymes or bacteria [48]; the most commonly employed fixative is formalin [49].

Similar to micro-CT imaging, staining is often employed to better distinguish the different biological structures; depending on the type of sectioned tissue under observation, different histology stains can be selected [47], depending on the selective affinity of the stain for different biological substances [50]. Since the majority of stains only absorb light, the stained histological slides are viewed using a light microscope [50], although electron microscopy can be used as well, but more rarely [47].

Slides can be stored and viewed years after their creation; however, in these cases, restaining and decoverslipping can be required [51]. Furthermore, light microscopic histology provides high-resolution images, but it is a destructive technique for the specimen and requires elaborate preparation [3]. Sometimes, it is difficult to perform quantitative analysis on histological samples due to inconsistencies in the preparation of histology slides [50]. Histological artifacts, such as autolysis artifact, formalin pigments, improper dehydration, knife lines, and residual wax, are related to the sample preparation procedures, which could impact the quantitative analysis performance (for a comprehensive list of histological artifacts, see [52]).

In conclusion, OM is an immediate imaging technique that can provide direct observation of the surface of the sample, giving a good perspective of geometry. It can be non-invasive; however, when the internal view is required, the destruction of the sample is inevitable.

### 2.3. Confocal Laser Scanning Microscopy

Confocal laser scanning microscopy (CLSM) is the most prevalent light microscopical technique used in biomedical sciences, utilising fluorescent probes for imaging complex and diverse three-dimensional structures. The basic concept of confocal imaging was initially introduced by Marvin Minsky in 1957 with the main ambition aiming to overcome the defects of the traditional fluorescence microscopes [53]. Ten years later, Eger and Petráň designed the first mechanical CLSM, which was actually a multiple-beam confocal microscope with a scanning disk (Nipkow disk) [54].

The main principle of CLSM is to generate an image consisted of fluorescence emitted by a specimen from a single plane of focus. This could be achieved by using a pinhole aperture that permits the filtering of the fluorescent signals, preventing the out-of-focus light from reaching the detector. Consequently, by regulating the size of the pinhole, the depth of the in-focus field in the sample can be determined [55].

Although conventional wide-field fluorescence microscopy is suitable for achieving high-resolution images (up to ≈200 nm laterally and 500 nm axially) in less complex structures, when it comes to thicker specimens (generally, anything thicker than 2 mm), images are often blurry and out-of-focus [56,57]. All the above along with the constant progress of computer, laser, and fluorescence technology in the late 1980s led eventually to the commercialisation of the instrument (CLSM) [58], which has since become one of the most valuable tools in biomedical research.

Several innovations that CLSM provides include the reduction or even elimination of out-of-focus light, the ability to control the depth of the field, and the generation of serial optical sections that can depict the three-dimensional structure of a thick specimen [56,59]. To this end, the architecture of the sample is preserved, since the instrument provides a non-invasive method of image construction using light, in contrast to methods in which the physical sectioning (e.g., via microtome) of specimens is required.

The resilience of the approach and the fact that compared to electron microscopy, CLSM requires remarkably less time for sample preparation, has made CLSM applicable in a broad range of studies and has extended its capabilities from imaging superficial tissues to the observation of cells’ and organs’ physiology [60,61]. Specifically, confocal imaging can be used to study cells with unique features such as neurons and cardiomyocytes. In addition, it can be applied in both living and fixed specimens [56,58], providing the ability to examine various dynamic processes, such as gene expression [62], mitochondrial dynamics and mobility, cytoskeletal assembly, and turnover, as well as molecule-molecule interactions and binding [63]. Moreover, incubation of samples with different fluorescent markers has given the opportunity not only to reveal position of proteins within the cell but also to analyse the co-localisation of protein expression patterns [64,65].

However, there are some defects to the CLSM system. More specifically, the laser power may cause sample photo-bleaching and attenuation of the signal [54]. Furthermore, there is a limited number of commercially available lasers regarding excitation wavelengths, and the point-by-point scanning (monofocal acquisition) is quite slow as well, providing few images per second [54].

Taken all together, confocal microscopy along with its modern advanced implementations has emerged as an indispensable microscopy technique for assessing networks of cellular components in both in vitro and in vivo studies. The continuous improvements of chemical and genetic labeling techniques, imaging instruments, and technologies, along with the development of new and successful disease animal models could lead to overcoming any drawbacks and deciphering a variety of pathological conditions, hence setting the future of modern medicine.

### 2.4. Scanning Electron Microscopy

Scanning electron microscopy (SEM) was invented building on the basic principles of light microscopy [66]. SEM is using a focused beam of high-energy electrons for the 2D and 3D characterisation of materials [67] and examination of microstructure morphology with high spatial resolution [66]. It scans the top 1 μm of the specimen, providing information on its topography, crystalline structure, and electrical behaviour [68]. Although light microscopy has been, and continues to be, of great importance to scientific research, it has a limit of resolution of 1000×, while SEM can reach magnifications up to 1,000,000× with an ultimate resolution of 1 nm or less [68].

The first electron beam scanner that was used on solid samples and had all the characteristics of an SEM was invented by Knoll [69]. A few years later, in 1938, the first Scanning Transmission Electron Microscope (STEM) was built with the ability to magnify an object up to 8000× and a resolution of 50–100 nm [70,71]. The first actual SEM was built a while later [72], and its general use started in 1965 [73].

The working principle of SEM is the following: (a) a confined and focused beam of high-energy electrons is emitted through the electron (thermal) source and accelerated towards the specimen using a positive electrical potential; (b) electrons interact with the atoms of the specimen, which in turn emit secondary electrons (SE) and backscattered electrons (BSE) electrons (signals); (c) signals are detected by the electron detector and transformed into an image [74].

Although SEM can be applied in various scientific fields and samples, and it can provide high-resolution images in a relatively short amount of time, there are certain limitations in the methodology. Conventional SEMs require high vacuum to operate in order to prevent gas molecules interfering with the electron beam and the SE and BSE. Thus, specimens that will be used in SEM must be dry and contaminant-free, which is highly problematic when dealing with biological and biomedical samples that will be, unavoidably, shrunk from drying [75]. However, there are certain methods, such as plunge and freeze drying that may preserve the native morphology of the sample [76] and cause milder shrinkage [77] and others that allow the examination of wet specimens without any prior preparation [78].

In addition, if non-metallic specimens are to be scanned, they need to be made conductive, either by sputter coating or vacuum deposition [79]. Commonly used metals to make the samples electrically conductive are gold, platinum, or palladium [75]. Thus, the preparation of biological and biomedical samples is a destructive procedure, and permissions are required if the samples are unique and/or rare, etc.

Overall, SEM is a valuable technique for offering relatively fast (1 to 2 days) and high-resolution images. However, its destructive nature is a factor that needs to be taken into account.

## 3. Case Studies

The biological and biomedical needs for three-dimensional and high-resolution images as well as for visualisation of the internal morphology of structures led to the use of the aforementioned imaging techniques in combination in order to exploit the advantages and at the same time overcome the disadvantages of each technique. Below, some case studies are presented where different combinations of these techniques have been used depending the scope of each study.

### 3.1. Biological

#### 3.1.1. Effects of Climate Change on Gastropod Shells and Egg Capsules

Shell calcification processes in marine organisms can be altered under low pH or increased temperature conditions [19,80,81]. Biogenic calcification rates are predicted to become lower than the CaCO_3_ dissolution rate as pH is reduced; thus, CaCO_3_ will be dissolving faster than it is being produced [82]. Morphological changes of the shell due to climate change can also subsequently affect the survival and behavior of gastropods. The species selected for the controlled experiments performed is the Neogastropod *Hexaplex trunculus* of the Muricidae family, which was experimentally subjected to combined conditions of warm temperature and increased seawater acidity. The increased temperature and the low pH conditions applied were representative of the high emissions (RCP 8.5) “business as usual” scenario of the Intergovernmental Panel on Climate Change models for eastern Mediterranean until the year 2100 [83].

**Figure 1:**
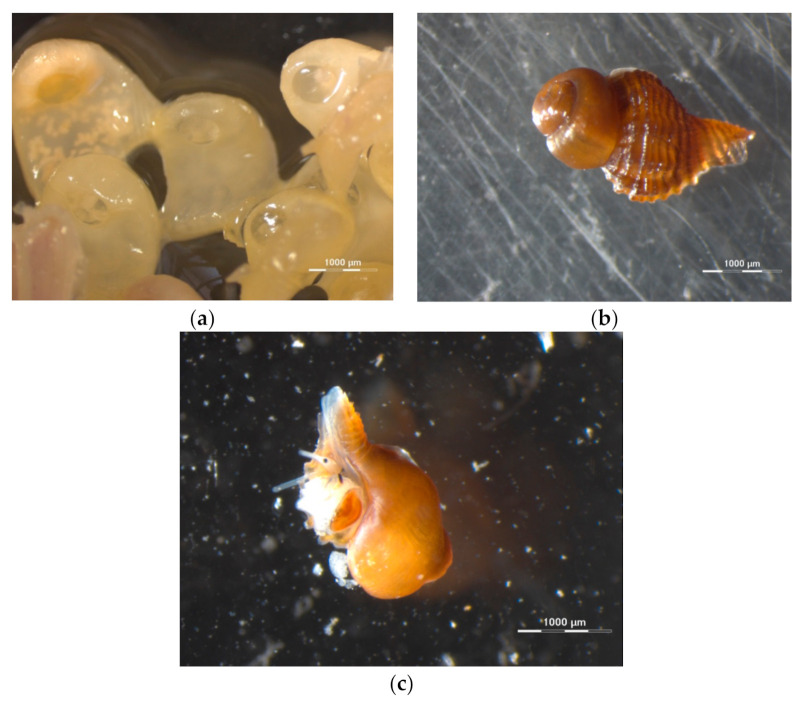
(**a**) Egg capsules (×8 magnification) and (**b**,**c**) living juveniles of *Hexaplex trunculus* (×25 magnification) using a stereoscope (Zeiss Discovery.V12).

The structure and the morphology of the gastropod shell and of the egg capsules of *H. trunculus* were studied using (a) the optical stereoscope and (b) the micro-CT techniques. The optical stereoscope (Zeiss Discovery.V12) was able to successfully and realistically demonstrate the outer surface of the shell and egg capsules, operating under simple and cost-effective terms (Figure 1a,b). Visualisation and size measuring of alive juveniles and egg capsules was also possible, since live photos could be obtained with camera shots under the stereoscope without sacrificing the specimens (Figure 1c). However, imaging of the full size of larger individuals or of the interior of the shell and egg capsules was not possible. The use of micro-CT, as an advanced 3D imaging and analytical technique, offered an additional insight in the internal shell microstructure, since it allowed measurements of specific architectural parameters of the full intact shell: shell density, structure thickness, and porosity.

**Figure 2:**
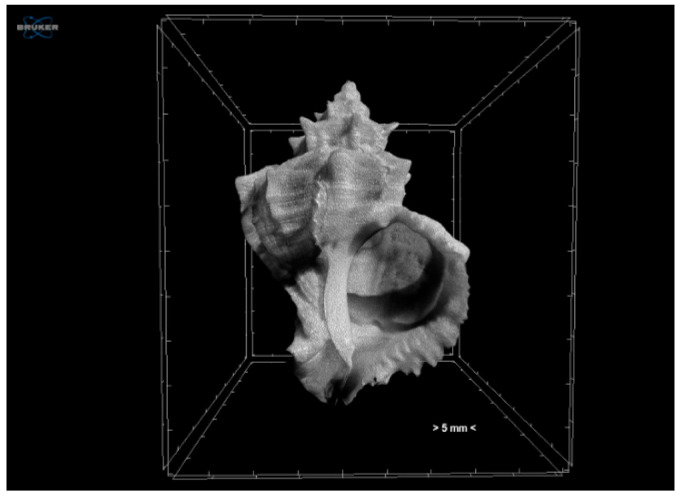
Volume rendering of an adult *Hexaplex trunculus* shell acquired using a Skyscan 1172 micro-CT scanner.

**Figure 3:**
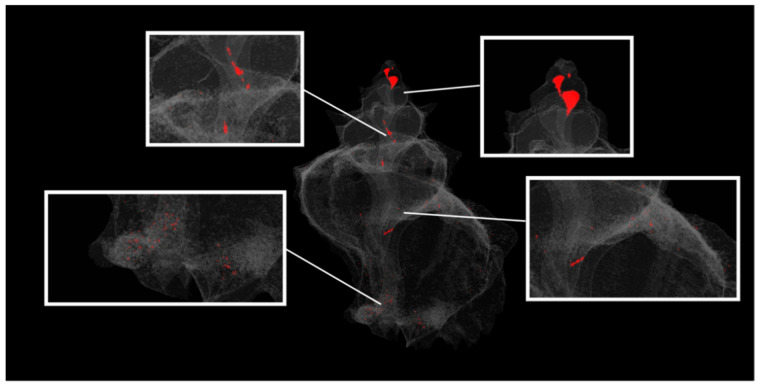
Three-dimensional (3D) model of the closed pores (indicated in red) of an adult *Hexaplex trunculus* shell (shell length 48.33 mm) acquired using a Skyscan 1172 micro-CT scanner.

**Figure 4:**
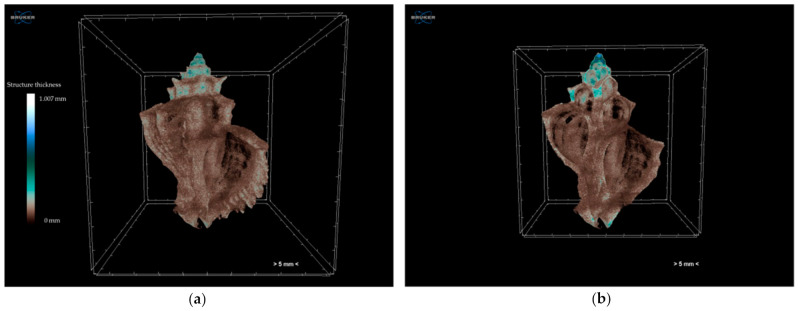
Colour-coded structure thickness images of (**a**) whole specimen and (**b**) cross-section along the specimen of an adult *Hexaplex trunculus* shell acquired using a Skyscan 1172 micro-CT scanner.

Adult shells were scanned without any staining at a voltage of 100 kV and a current of 100 μA using a combined aluminium and copper filter with a SkyScan 1172 micro-CT (Bruker, Kontich, Belgium). Images were acquired at a pixel size of 13.79 μm with a camera binning of 2 × 2. Exposure time was 2480 ms, and scans were performed for a half-rotation of 180° and a rotation step of 0.60° in order to minimise the scanning duration. Projection images were reconstructed into cross-sections using SkyScan’s NRecon v.1.7.4.2 software (Bruker, Kontich, Belgium) in a range of attenuation coefficients of 0–0.13, with a beam-hardening correction of 59%, smoothing of 2, and ring artifact correction of 20. The reconstructed images were stored as 16-bit TIFF images. Volume renderings of each specimen were created using the SkyScan’s CTVox v.3.3.1 software (Bruker, Kontich, Belgium) in order to display the reconstructed images as a 3D object (Figure 2). The 3D analysis tools offered with the micro-CT technique (SkyScan’s CTAn v.1.18.4.0 software, Bruker, Kontich, Belgium)) enabled (a) the calculation of mean greyscale values of the total shell as a proxy of the relative density of the calcified tissues, (b) the calculation of porosity (Figure 3), and (c) the estimation of the 3D structure thickness in each specimen (Figure 4). Furthermore, 3D geometric morphometric methods can be applied for shape comparisons between different specimens through the creation of surface models and the application of specified landmarks on them. The estimation of the above-mentioned characters can be valuable for the investigation of potential morphological and operational alterations of the shell due to the experimental effects of warmer temperature and low pH.

**Figure 5:**
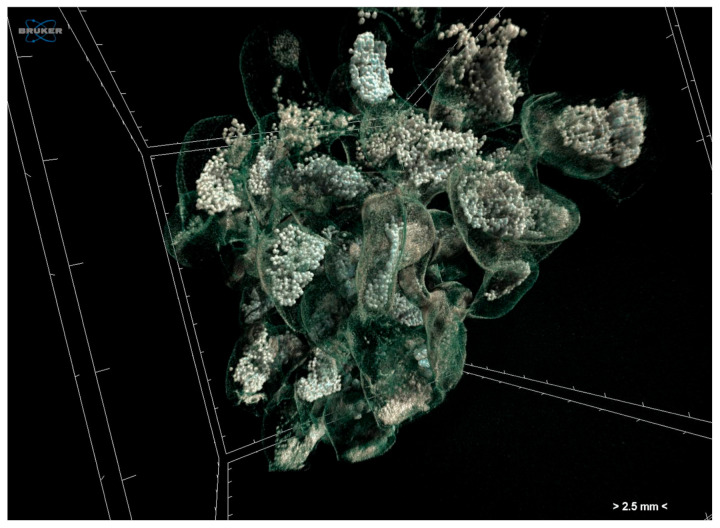
Volume rendering of egg capsules of *Hexaplex trunculus* acquired using a Skyscan 1172 micro-CT scanner.

Egg capsules of *H. trunculus* were fixed in 5% formaldehyde and stained with 1% iodine in 96% ethanol modifying the staining protocol included in Metscher [23]. Specimens were scanned at a voltage of 80 kV and a current of 124 μA using an aluminium filter. Scanning was performed in 96% ethanol as a scanning medium. Images were acquired at a pixel size of 13.79 μm with a camera binning of 2 × 2. The exposure time was 1435 ms, and scans were performed for a full rotation of 360° and a rotation step 0.40°. Projection images were reconstructed into cross-section images in a range of attenuation coefficients of 0–0.064, with a beam-hardening correction of 59%, smoothing of 2, and ring artifact correction of 20. The reconstructed images were stored as 16-bit TIFF images. Micro-CT had the ability to visualise the interior structure of the egg capsules, the capsule walls, and the numerous eggs they contain (Figure 5).

**Figure 6:**
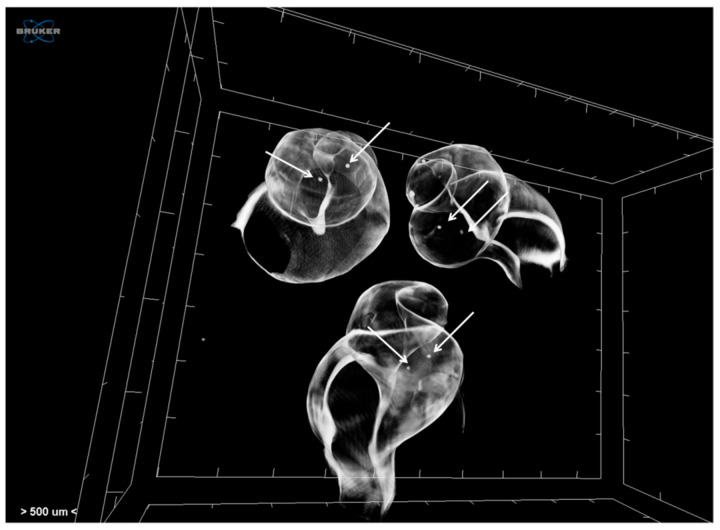
Volume rendering of juvenile *Hexaplex trunculus* acquired using a Skyscan 1172 micro-CT scanner, where internal calcified structures (statoliths, indicated with white arrows) are also visible.

Juveniles of *H. trunculus* were also scanned after anesthetisation with 7% MgCl_2_ and without any staining in order to visualise their shell properties (similarly to adults). Specimens were scanned at a voltage of 59 kV and a current of 167 μA without any filter. Images were acquired at a pixel size of 2 μm with a camera binning of 1 × 1. Exposure time was 325 ms, and scans were performed for a full rotation of 360° and a rotation step 0.20°. Projection images were reconstructed into cross-section images in a range of attenuation coefficients of 0–1.127, with a beam-hardening correction of 59%, smoothing of 2, and ring artifact correction of 20. The reconstructed images were stored as 16-bit TIFF images. Micro-CT imaging has also enabled the visualisation of their interior calcified structures (i.e., statoliths), which otherwise would require laborious and time-consuming operations in order to achieve their manual extraction (Figure 6).

#### 3.1.2. Taxonomic Identification of Polychaeta

Polychaetes are one of the most abundant benthic taxa either in hard or in soft bottom marine habitats [84]. The systematic identification of this taxon is problematic in many cases due to literature gaps, based on characters that are difficult to distinguish. More specifically, polychaete taxonomy is using internal structures such as jaws, glands, and pharyngeal structures, as well as external ones such as chaetae, parapodia, and antennas as key taxonomic characters. Therefore, there are cases in which it is important to visualise the animal internally and at the same time to keep it intact. Thus, a combination of imaging techniques is often required in order to identify a specimen and describe a new species.

Optical microscopy is the most common technique for this process; however, SEM and micro-CT are also used either for better results or due to their specific imaging features. In the literature, the majority of studies describing new species is using a combination of OM and SEM (e.g., [85,86]).

**Figure 7:**
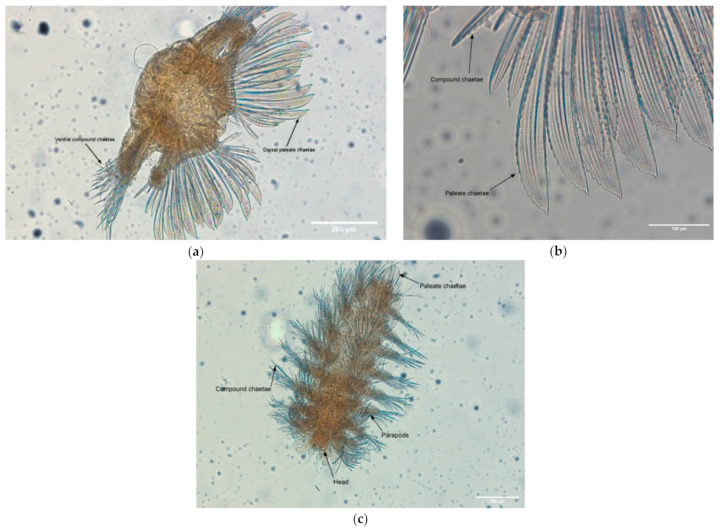
OM images of (**a**) middle body cross-section of a specimen showing the orientation and the type of chaetae (×40 magnification), (**b**) detailed view of middle body chaetae (×60 magnification), and (**c**) body shape of the specimen (×40 magnification).

The Natural Geography in Shore Areas project (NaGISA, http://www.coml.org/natural-geography-shore-areas-nagisa/) aimed at inventorying and monitoring coastal habitat-specific biodiversity, globally. In the framework of the NaGISA, new polychaete species collected from coastal rocky substrates from the island of Crete have been described using OM, micro-CT, and SEM. For OM, specific body parts were dissected from the specimen in order to reveal the key characters of the species (Figure 7).

**Figure 8:**
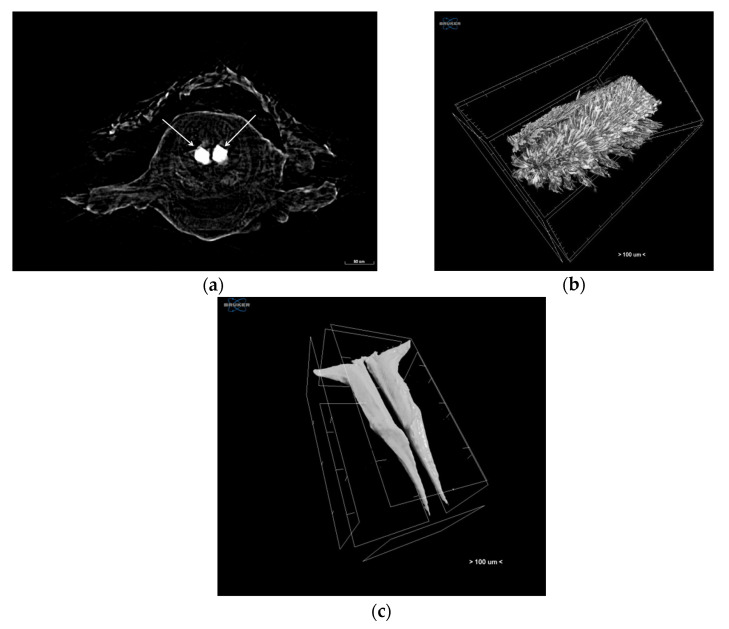
Micro-CT images of (**a**) cross-section of the middle body of specimen, with white arrows showing the jaws, (**b**) body shape of the specimen, and (**c**) jaws of the specimen.

Micro-CT was chosen since it can be used to visualise the external and the internal structure of species. For this study, polychaetes were scanned without any staining at a voltage of 59 kV and a current of 167 μA without filter with a SkyScan 1172 micro-CT (Bruker, Kontich, Belgium). Images were acquired at a pixel size of 0.98 μm with a camera binning of 1 × 1. Exposure time was 1915 ms, and scans were performed for a full rotation of 360° and a rotation step of 0.25°. Projection images were reconstructed into cross-sections using SkyScan’s NRecon software in a range of attenuation coefficients of 0.033–0.43, with a beam-hardening correction of 20%, smoothing of 2, and ring artifact correction of 10. The reconstructed images were stored as 8-bit PNG images. Volume renderings of each specimen were created using the SkyScan’s CTVox software (Figure 8).

**Figure 9:**
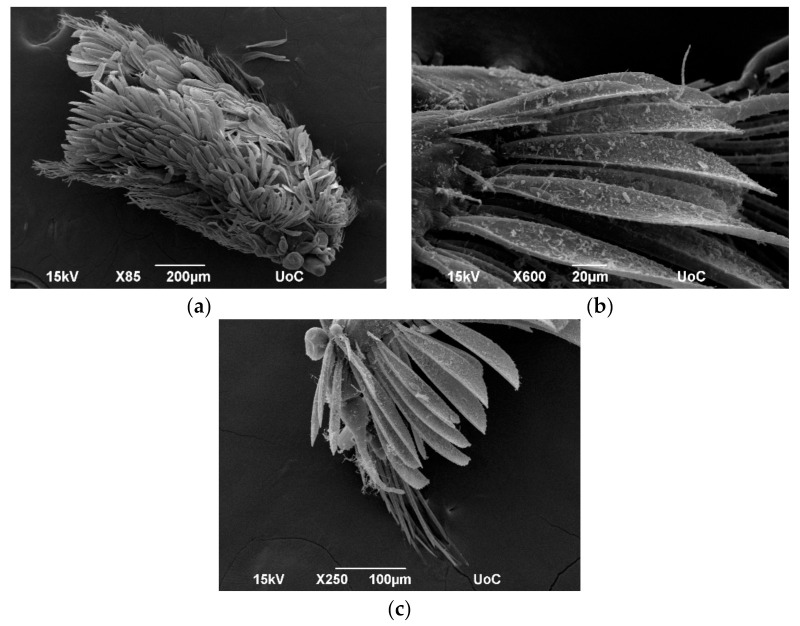
SEM images of (**a**) body form of specimen, (**b**) 3D form of paleae chaete, and (**c**) shape of paleae chaete and compound chaete of middle body.

For SEM, samples were fixed in 98% ethanol followed by dry ethanol on room temperature twice 20 min/time. Dehydrated samples were critical point dried (Baltec CPD 030) and mounted on stubs prior to sputter coating with 20 nm thick gold (Baltec CPD 050). Observation was carried out using a SEM (JEOL, model JSM-6390LV, Jeol USA Inc, Peabody, MA, USA) at 15 kV operating voltage. As mentioned in Section 2, SEM images are only surface images (Figure 9), and since the samples were sputtered with gold, they could not have been used for any other technique after this process.

Optical microscopy has a very thin focus area; thus, cross-sections are preferred, in contrast with SEM where the focus range is much greater. In addition, OM does not require pre-treatment and is able to capture the true colours of the specimen. Furthermore, it is a fast and relatively cheap way to create images, with no limitations for shape or geometries of the examined objects. However, due to the low resolution of OM, other imaging techniques were also applied for this study.

Micro-CT had the ability to produce images from the outer and the inner structures of the specimen but with low resolution (limited to a few mm) and focus depth. On the other hand, SEM produced high-resolution images with great focus depth but at the cost of specimen destruction.

Since a single method to produce images for the description of new species does not exist, a combination of the aforementioned techniques is required to achieve the best results.

### 3.2. Biomedical

#### 3.2.1. Evaluation of Extracted Thrombotic Material Characteristics

Micro-CT imaging was utilised in an effort to evaluate the impact of aspirated thrombus burden on the post-aspiration clinical and angiographical outcomes of patients with ST-Elevated Myocardial Infarction (STEMI) [13]. The prognostic significance of initial angiographic and post-aspiration residual thrombus burden has been already investigated via recent optical coherence tomography studies [87]. However, this study, which employed micro-CT, aimed to fill the gap of evidence on the quantification of extracted thrombotic material characteristics. Up to date, these characteristics have been subjective. Therefore, since micro-CT allows non-destructive 3D imaging of both the internal and external microstructure of the samples studied, it was deemed to be the most qualified to bridge this evidence gap. In particular, researchers sought to characterise and assess for the first time extracted thrombotic material characteristics (volume, surface, and density) via micro-CT in 113 patients with STEMI undergoing manual aspiration thrombectomy [88].

**Figure 10:**
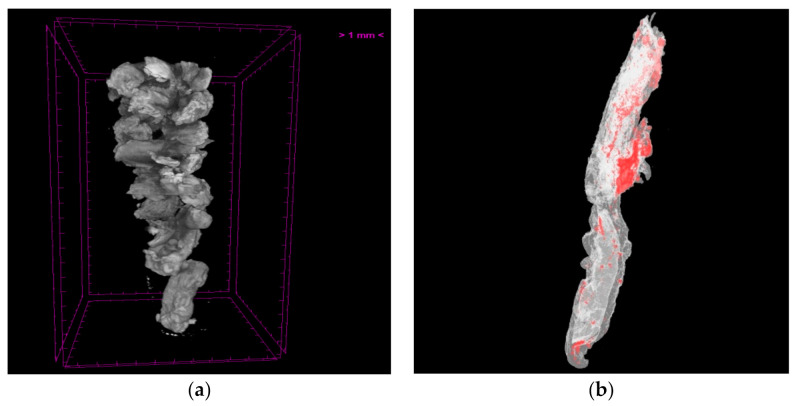
(**a**) Volume rendering of thrombi and (**b**) 3D color visualisation of a thrombus sample (volume 16 mm^3^) acquired through the Skyscan 1172 micro-CT scanner: Erythrocyte-rich regions were rendered in red, whereas platelet-rich regions were rendered in white. (**b**) from [13], reproduced under a CC-BY licence.

**Figure 11:**
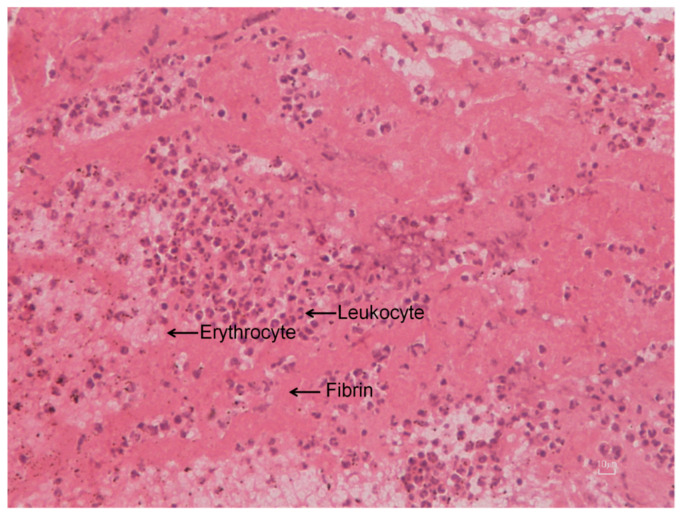
OM image (×200 magnification) of a histological examination of a thrombotic specimen revealed a thrombus characterised by fibrin/erythrocytes (occupied 70% of the total area of the thrombus) and leukocytes (30%).

Extracted thrombotic material was initially preserved in 10% formalin for 24 h and subsequently dehydrated in graded series of ethanol solutions up to 70%. In order to strengthen the tissue contrast and achieve precise micro-CT 3D imaging of both the internal and external microstructure of the clots, 0.3% phosphotungstic acid (PTA) in 70% ethanol was used as a contrast agent according to the staining protocol presented in Metscher [23]. Micro-CT scans were performed with a SkyScan 1172 micro-CT (Bruker, Kontich, Belgium) at a voltage of 48 kV and a current of 204 μA without filter. Images were acquired at a pixel size of 4 μm with a camera binning of 1 × 1. Exposure time was 325 ms, and scans were performed for a full rotation of 360° and a rotation step of 0.25°. The projection images were reconstructed into cross-sections using SkyScan’s NRecon software in a range of attenuation coefficients of 0–0.7, with a beam-hardening correction of 59%, smoothing of 2, and ring artifact correction of 20. The reconstructed images were stored as 16-bit TIFF images. Cross-section images were loaded into the software CT Analyser v.1.14.4.1 (CTAn, Bruker, Kontich, Belgium) to extract measurements for the volume and density of thrombi as mean greyscale values (±Standard Deviation), which were also converted to Hounsfield units (HU). Furthermore, erythrocyte-rich and platelet-rich regions of the thrombus were segmented through the CTAn software using different grey-level values (for more details, see [13]). Finally, volume renderings of the samples were created using the SkyScan’s CTVox software (Figure 10a). Skyscan’s CTVol v.2.3.2.0 software was also used for the creation of a 3D model that can display the erythrocyte-rich and platelet-rich regions of the thrombus (Figure 10b). Apart from the volumetric thrombotic characteristics derived (mean extracted thrombus volume, surface, and density were 15.71 (±20.10) mm^3^, 302.89 (±692.54) mm^2^, and 3139.04 (±901.88) HU, respectively), the clot relative density was also calculated and, thus, offered the opportunity to quantify the presence of different cell types (erythrocytes, leukocytes, and platelets) within the clot (Figure 10b). Histological examination was also performed to complement the micro-CT analysis, and it revealed features of lytic thrombus after staining the formalin-fixed paraffin-embedded specimen with hematoxylin and eosin (Figure 11).

This analysis demonstrated that micro-CT analysis of thrombotic specimens was feasible, since thrombotic material characteristics were effectively quantified in all thrombi with no sample disintegration observed. Micro-CT scanning was also reliable and reproducible, having great intra- and inter-observer reliability (interclass correlation coefficients for thrombus volume, surface, and density were equal to 0.995 (95% C.I.: 0.981–0.998), 0.995 (95% C.I.: 0.991–0.996), and 0.987 (95% C.I.: 0.966–0.993), respectively) [13]. The correlation of micro-CT images with clinical and angiographical parameters yielded that larger thrombus burden was associated with outcomes suggestive of poor prognosis in patients with STEMI, since higher thrombus volume and surface were independently associated with distal embolisation (*p* = 0.007 and *p* = 0.028, respectively), no-reflow phenomenon (*p* = 0.002 and *p* = 0.006, respectively), and angiographically evident residual thrombus (*p* = 0.007 and *p* = 0.002, respectively) [13].

Although micro-CT facilitates the non-destructive volumetric acquisition of high-resolution images at near-histological spatial resolution [89], large radiation doses of micro-CT scanning constitute the main restriction of the technique, limiting its clinical application for in vivo clinical trials, even if some studies have already performed in vivo imaging in animal models. Moreover, in this study, specimen preservation in formalin solutions and staining with PTA might have altered thrombus measurements, as the specimen may have been affected by shrinkage or other alterations due to the acidic nature of the staining [20,90], and, therefore, there is still some distance to be covered in order to utilise micro-CT scanning in clinical trials involving soft tissues in vivo [91].

Nevertheless, QUEST-STEMI (Quantification of Extracted Thrombus Burden Characteristics and Association With Angiographic Outcomes in Patients With ST-Elevation Myocardial Infarction) is the first study of volumetric coronary thrombus assessment by micro-CT, and the observed scanning protocol could be used in other clinically-oriented trials in future to help to achieve personalised risk stratification-based care management, in accordance with thrombus burden encountered in each individual [92].

#### 3.2.2. Cardiotoxicity Due to Proteasome Dysfunction

Multiple myeloma (MM) is a plasma cell disease, the second most common hematologic malignancy in the USA [93], and it is considered a treatable but generally incurable cancer type [94]. It is characterised by the uncontrolled growth of monoclonal plasma cells in bone marrow, resulting in the overproduction of fractions or non-functional intact immunoglobulins.

Proteasome inhibitors (PIs), such as bortezomib (a slowly reversible PI) or carfilzomib (binds irreversibly to proteasome), are indicated in the treatment of MM and mantle cell lymphoma and are under evaluation for the treatment of other malignancies [95]. PIs target the ubiquitin–proteasome system, which plays a key role in maintaining normal cellular homeostasis in eukaryotic cells, hence regulating the majority of intracellular proteins [96]. Specifically, carfilzomib has demonstrated clinical efficacy in patients with relapse or refractory MM [97]. Nevertheless, treatment with carfilzomib has been associated with a significant incidence of cardiotoxicity, especially in patients with known cardiovascular risk factors [98].

**Figure 12:**
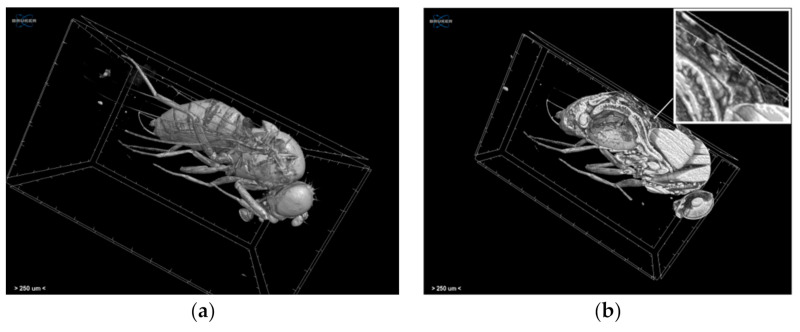

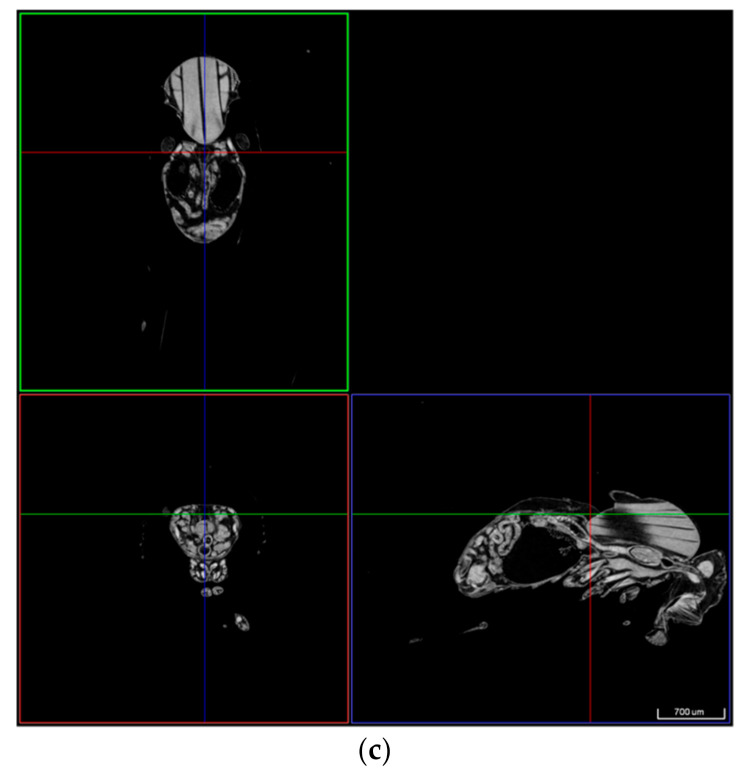
Volume rendering of (**a**) the whole specimen and (**b**) the virtually dissected specimen and (**c**) transaxial, sagittal, and coronal images of the *Drosophila melanogaster* acquired through the Skyscan 1172 micro-CT scanner. The white arrow and the cross-point of the coloured lines indicate the heart position.

By exploiting the *Drosophila melanogaster* as an in vivo model [95], cardiac toxicity caused by proteasome dysfunction can be examined using various imaging techniques including CLSM and micro-CT in order to study possible deterioration of the heart functionality and structure. Specifically, micro-CT technology can be applied to visual evaluate features of *Drosophila*’s heart, thus providing both qualitative and quantitative data. *Drosophila*’s specimens were preserved in 4% formalin solution, and they were later subjected to dehydration procedures and stored in alcohol solution. In order to strengthen the tissue contrast, 1% iodine dissolved in 96% ethanol was used as a contrast-agent modifying the staining protocol presented in Metscher [23]. Micro-CT scans were performed with a SkyScan 1172 micro-CT (Bruker, Kontich, Belgium) at a voltage of 50 kV and a current of 198 μA without filter. Images were acquired at a pixel size of 2.9 μm with a camera binning of 1 × 1. Exposure time was 316 ms, and scans were performed for a half rotation of 180° and a rotation step of 0.23°. Projection images were reconstructed into cross-sections using SkyScan’s NRecon software in a range of attenuation coefficients of 0–0.447611, with a beam-hardening correction of 59%, smoothing of 2, and ring artifact correction of 20. The reconstructed images were stored as 16-bit TIFF images. Volume renderings of each specimen were created using the SkyScan’s CTVox software in order to display the reconstructed images as a 3D object (Figure 12a,b). SkyScan’s DataViewer v.1.6.5.2 software was used in order to explore the cross-section images (Figure 12c).

**Figure 13:**
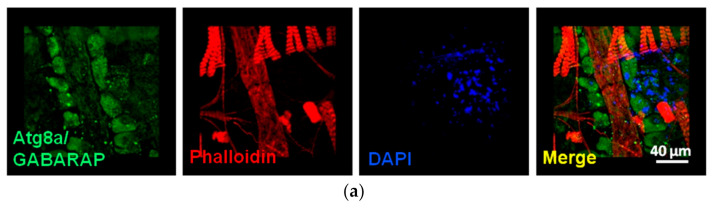

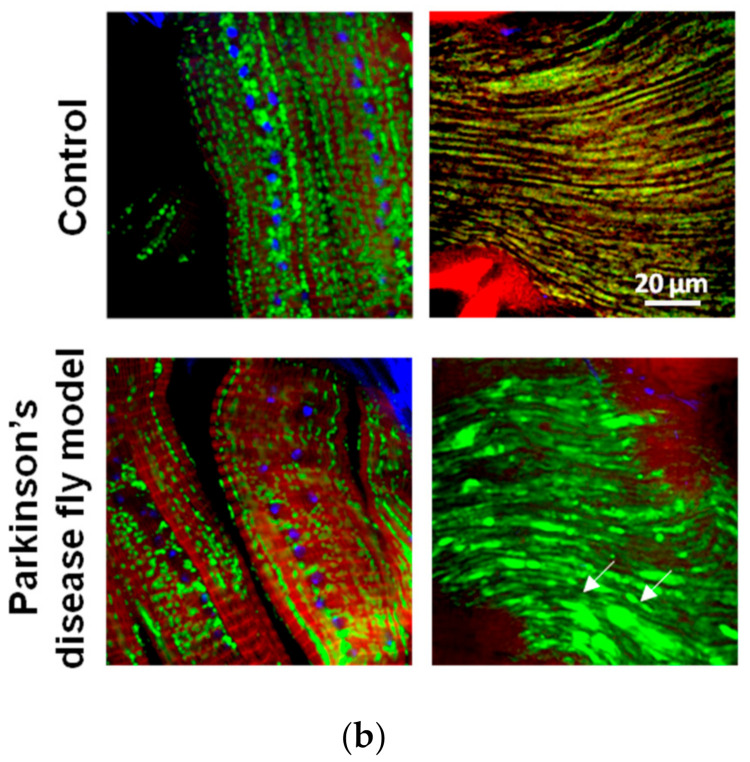
CLSM visualisation (×60 magnification) of different stained *Drosophila melanogaster* tissues. (**a**) *Drosophila* heart tissue following immunofluorescence staining with an antibody against the Atg8a/GABARAP autophagic protein. (**b**) Different sections of a fly muscle probed with the mitochondrial marker blw/ATP5A (green) in a Parkinson’s disease fly model, showing accumulation of mitochondrial aggregates (arrows) vs. control. In (**a**,**b**) nuclei and actin were counterstained with DAPI and phalloidin, respectively. In (**a**), Z-stacks with a step size of 1 µm were taken using identical settings, and each stack consisted of 26 plane images.

CLSM analysis can also be used to examine the structural differences between the control and flies treated with PIs, choosing probes such as phalloidin, which is a highly selective bicyclic peptide that is used for staining actin filaments. In addition, several commercially available antibodies against various cellular components can also be administrated to *Drosophila* heart to analyse fluctuations in protein expression patterns and to study possible effects on organelles such as mitochondria and lysosomes not only in cardiac disease models but also in neurodegenerative models (i.e., Parkinson’s disease) (Figure 13) or in other pathological conditions (i.e., aging). Specifically, young flies were dissected, and cardiac tubes or muscle tissues were isolated in PBS and then fixed in 4% formaldehyde for 15 min and washed in PBS. *Drosophila*’s tissues were incubated with primary antibodies against the protein of interest and subsequently with secondary antibodies, DAPI [nuclei staining (4′,6-diamidino-2-phenylindole)], and/or phalloidin for 1 h at RT. Visualisation of samples was done by using a Digital Eclipse C1 Nikon (Melville, NY, UAS) CLSM equipped with 60 × 1.40 NA DIC Plan Apochromat objectives, using the EZC1 acquisition and analysis software (Nikon, Minato City, Tokyo, Japan).

Using micro-CT in this biomedical study, data from several structures of *Drosophila melanogaster*’s cardiac tube were obtained, avoiding compromising the tissues’ features. In this manner, morphological alterations from specific domains of the heart could be detected in intact animals. Notably, dimensions of the heart tube and measurements such as heart density and structure thickness were assessed and revealed differences comparing control vs. flies exposed to PIs. Despite the non-destructive approach of micro-CT technology, molecular and cellular interactions in these types of studies, as mentioned before, should also be taken into consideration and not be excluded. Therefore, these observations combined with data from CLSM analysis, targeting specific proteins in the heart, were used in order to determine the extent of cardiotoxicity.

#### 3.2.3. Craniosynostosis in Mice Due to Erf Insufficiency

Craniosynostosis, the premature ossification of the cranial sutures, is a potentially lethal disease affecting approximately 1 in 2500 newborns [99]. Recent data indicate that ERF haploinsufficiency leads to craniosynostosis (CRS-4) in both humans and mice [100]. ERF is a transcriptional repressor member of the ETS family of transcription factors that is inactivated by the FGF/RAS/ERK pathway (Fibroblast growth factors/Fibroblast growth factors/extracellular signal-regulated kinase) via phosphorylation and nuclear export [101]. Pharmacological factors that augment ERF suppressor activity were tested in the mouse model to assess their ability to ameliorate the ERF-related craniosynostosis (to be published elsewhere). On postnatal day 65 (P65), the animals were sacrificed and skull development, suture closure, calvarial bone thickness, and morphology were analysed by micro-CT.

**Figure 14:**
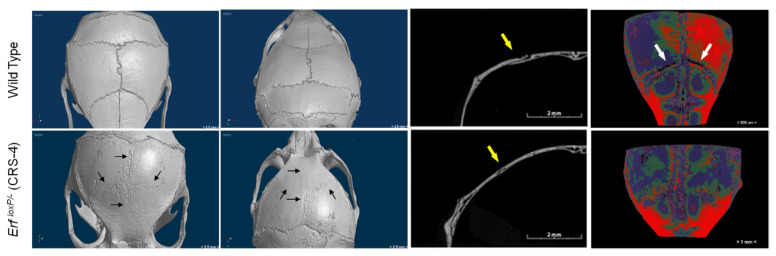
(**a**) Three-dimensional (3D) volume renderings, (**b**) transverse section image midway through the right coronal suture, and (**c**) bone thickness visualisation derived from microCT scans of wild-type and a *Erf^loxP/−^* (CRS-4) animal with multisuture synostosis at P65. Black arrows indicate the fused sutures. Yellow arrows point to the coronal suture position in the transverse sections. White arrows indicate the minimal bone thickness in the coronal suture region in the wild-type animal.

Micro-CT technology seems to be a useful tool for visual evaluation of skulls with multisuture synostosis. Mouse skulls were scanned without any staining at a voltage of 75 kV and a current of 131 μA using an aluminium filter with a SkyScan 1172 micro-CT (Bruker, Kontich, Belgium). Images were acquired at a pixel size of 13.79 μm with a camera binning of 2 × 2. Exposure time was 1435 ms and scans were performed for a half rotation of 180° and a rotation step 0.40°. Projection images were reconstructed into cross-sections using SkyScan’s NRecon software in a range of attenuation coefficients of 0–0.089961, with a beam-hardening correction of 59%, smoothing of 2, and ring artifact correction of 20. The reconstructed images were stored as 16-bit TIFF images. Volume renderings of each specimen were created using the SkyScan’s CTVox software in order to display the reconstructed images as a 3D object. Facial deformities and fused sutures on the skull of the *Erf^loxP/−^* mouse were revealed (Figure 14). Furthermore, qualitative and quantitative results can be derived by the micro-CT, as differences in the calvarial bone thickness were observed with thinner structures in the *Erf^loxP/−^* mouse compared to the wild type (Figure 14c).

**Figure 15:**
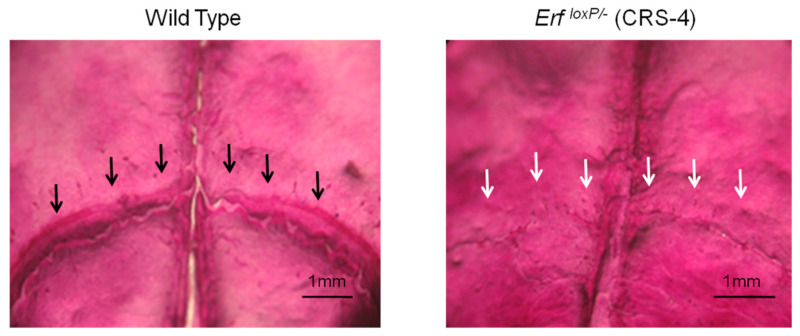
OM image (×1 magnification) of a histological examination of the coronal sutures of P65 mouse calvaria stained with Alizarin Red and Alcian Blue. Black arrows indicate the position of the open suture in wild-type animals and white arrows indicate the position of the ossified suture in the *Erf^loxP/−^* (CRS-4) craniosynostosis animals.

Similarly, histological examination of skulls after Alizarin Red S and Alcian blue staining, under stereoscopic light microscopy on a Leica MZ12 (Wetzlar, Germany), indicate the position of the open coronal sutures in wild-type animals and the position of the respective ossified sutures in craniosynostosis animals, while the sagittal suture is visible in both animals (Figure 15). Histological examination can provide additional information regarding heterogeneous structures stained by Alcian blue; however, it requires considerably longer manipulation time and effort and cannot display the 3D morphology of the coronal sutures of the skulls.

**Figure 16:**
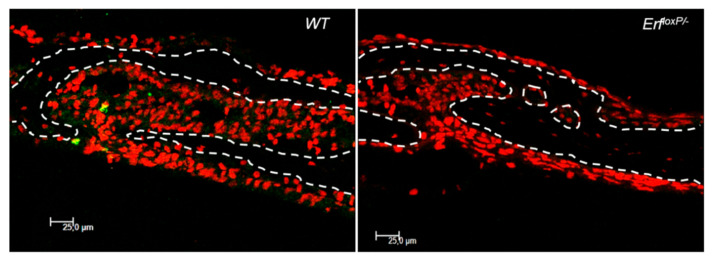
Confocal microscopy (×63 magnification) of coronal sutures transverse cryosections from P15 mouse calvaria stained with BrdU (green) to evaluate cellular proliferation and TOPRO-3 (red) to identify the nuclei. Dotted lines indicate the position of the parietal and frontal bones.

Confocal microscopy on a Leica SP8 scanhead fitted on a Leica DMi8 microscope (Wetzlar, Germany) of skull sections in the sutures was also used to evaluate changes at the cellular level as proliferation, apoptosis, or cell type. For example, the *Erf^loxP/−^* craniosynostosis animals exhibited decreased mesenchymal space and absence of proliferating cells as early as postnatal day 15 (P15) (Figure 16). This method is suitable for studying cellular and molecular events in situ, and possibly, it can illuminate underlying mechanisms. However, it is considerably more laborious and time consuming, and it cannot demonstrate changes affecting the sutures and the craniofacial morphology and ultimately the disease severity.

## 4. Conclusions

In Table 1, the imaging properties of each case study as well as the advantages and disadvantages of each technique are summarised. Micro-CT is an imaging technique that brought revolutionary changes in biological and biomedical research, taking advantage of its ability to create 3D morphological and anatomical data with great resolution. It can depict the internal structure of the sample, offering reliable measurements of a series of characters such as volume, density, and porosity. Its non-destructive nature enables the scanning of rare and valuable samples, as well as of samples that need to be further processed by other methods, providing also the possibility to repeat the analysis. However, micro-CT also has several limitations such as the potential alteration of the specimen characteristics due to the use of staining and the time needed to complete high-resolution scans. The limitations may be overcome by using other imaging techniques. OM can overcome the restrictions caused by the pre-treatment of the samples and the time needed, as the examination of the specimens can be immediate. Moreover, the specimens can be observed in their original form. However, the technique suffers by thin focus area. In addition, the samples are destroyed in cases where internal observation is needed. CLSM, although another OM technique, offers visualisation of the cellular and molecular level. Pre-treatment of samples is fast, and the technique gives the opportunity to study samples also *in vivo*. Despite the advantages, there is limited availability regarding the necessary antibodies, and laser power entails the risk of artifacts by causing photo-bleaching. SEM can offer very detailed images as it has very high resolution with great focus of depth. Nevertheless, the technique has high cost due to the necessary pre-treatment of the samples and offers only surface images.

The selection of the most appropriate technique depends on the scope of the research and the imaging technical features. The combination of 2D and 3D imaging techniques as complementary data can be the key element for a more comprehensive approach in biological and biomedical studies. Technology is continuously advancing in the field to respond to the research needs and exceed all the possible drawbacks. The way forward seems to be the integration of the results of different imaging techniques into the same outcome: towards more complete image evidence emerging from the samples scanned.

## Figures and Tables

**Table 1:** Brief description of the case studies presenting the imaging properties and the advantages and disadvantages of each technique.

		Biological		Biomedical			Advantages	Disadvantages
		Case Study 1 (Section 3.1.1)	Case Study 2 (Section 3.1.2)	Case Study 3 (Section 3.2.1)	Case Study 4 (Section 3.2.2)	Case Study 5 (Section 3.2.3)		
Micro-CT	pixel size (μm)	2–13.79 μm	0.98 μm	4 μm	2.9 μm	13.8 μm		
	sample preparation	only for egg capsules (fixation and staining—4 days)	no	yes (fixation and staining—2 days)	yes (fixation and staining—2 days)	no	internal specimen microstructure, measurements of specimen characteristics (volume, surface, density, thickness, porosity), reliable, reproducible, non-destructive	time consuming for high-resolution datasets, staining might alter specimen characteristics, large dataset size
	visualisation of features	external and internal shell and egg structures	external and internal structure	thrombotic characteristics	heart	facial deformities and fused sutures on the skull		
	destructive	potential alterations in egg capsules due to staining procedure	no	potential alterations in thrombotic measurements due to staining procedure	potential alterations in heart structure due to staining procedure	no		
	imaging duration	2.5–6 h	3 h	1.5 h	2 h	2 h		
OM	magnification	8–25×	40–60×	200×		1×		
	sample preparation	no	no	yes (fixation and staining—1 day)		yes (fixation, staining and removal of remaining tissues—35–40 days)	no pre-treatment, original colours of the specimen, fast if no sample preparation is needed, no limitations for shape or geometries, application to in vivo trials, non-invasive	very thin focus area, destruction of the sample in case that the internal view is needed, time consuming for histological examination
	visualisation of features	only external shell and egg structures	external structure	presence of different cell types		sutures		
	destructive	no	yes (cross-sections from specific parts)	yes		yes		
	imaging duration	immediate	immediate	immediate		immediate		
CLSM	magnification				60×	63×		availability of antibodies, photo-bleaching by laser power, destructive method
	sample preparation				yes (fixation, incubation with antibodies—2.5 h)	yes (fixation, incubation with antibodies—6–7 days)	study of organelles, visualisation at the cellular level, studying cellular and molecular events, can be applied to in vivo studies	
	visualisation of features				organelles in selected tissues	proliferation, apoptosis, and cell type		
	destructive				yes (young flies were dissected, cardiac tubes or muscle tissues were isolated)	yes		
	imaging duration				immediate	4–6 h		
SEM	magnification		85–600×					
	sample preparation		yes (dehydration and sputter coating with 20 nm thick gold—1 day)				High-resolution images with great focus depth	only surface images, destructive method
	visualisation of features							
	destructive		yes (fixation, dehydration, sputtering)					
	imaging duration		immediate					

## Data Availability

The datasets generated during and/or analysed during the current study are available from the corresponding author on reasonable request.
